# The addition of mobile SMS effectively improves dengue prevention practices in community: an implementation study in Nepal

**DOI:** 10.1186/s12913-019-4541-z

**Published:** 2019-10-15

**Authors:** Ashmin Hari Bhattarai, Guardian Yoki Sanjaya, Anil Khadka, Randeep Kumar, Riris Andono Ahmad

**Affiliations:** 1 International Master Programme in Public Health, Faculty of Medicine, Public Health and Nursing, Universitas Gadjah Mada, Yogyakarta, Indonesia; 2 Department of Health Policy and Management, Faculty of Medicine, Public Health and Nursing, Universitas Gadjah Mada, Yogyakarta, Indonesia; 3 Department of Public Health, Nobel College, Kathmandu, Nepal; 4 Department of Biostatistics, Epidemiology and Public Health, Faculty of Medicine, Public Health and Nursing, Universitas Gadjah Mada, Yogyakarta, Indonesia; 5 Centre for Tropical Medicine, Faculty of Medicine, Public Health and Nursing, Universitas Gadjah Mada, Yogyakarta, Indonesia

**Keywords:** Implementation research, Effectiveness, Acceptability, Appropriateness, Dengue prevention, Mobile SMS, Nepal

## Abstract

**Background:**

Dengue is an emerging vector disease with frequent outbreaks in Nepal that pose a major threat to public health. Dengue control activities are mostly outbreak driven, and still lack systematic interventions while most people have poor health-related knowledge and practices. Mobile Short Message Service (SMS) represents a low-cost health promotion intervention that can enhance the dengue prevention knowledge and practices of the affected communities. This study aimed to explore the acceptability, appropriateness, and effectiveness of mobile SMS intervention in improving dengue control practices.

**Methods:**

This study was an implementation research that used mixed-methods design with intervention. A total of 300 households were divided into three groups, i.e. one control group, one dengue prevention leaflet (DPL) only intervention group and one DPL with mobile SMS intervention group (DPL + SMS). We used a structured questionnaire to collect information regarding participants’ knowledge and practice of dengue prevention. We conducted in-depth interviews with key informants to measure acceptability and appropriateness of intervention. Mean difference with standard deviation (SD), one-way ANOVA, paired t-test and regression analyses were used to assess the effectiveness of the interventions. Thematic analysis was used to assess the acceptability, and appropriateness as well as barriers and enablers of the intervention.

**Results:**

The DPL + SMS intervention produced significantly higher mean knowledge difference (32.7 ± 13.7 SD vs. 13.3 ± 8.8 SD) and mean practice difference (27.9 ± 11.4 SD vs 4.9 ± 5.4 SD) compared to the DPL only group (*p* = 0.000). Multivariate analysis showed that the DPL + SMS intervention was effective to increase knowledge by 28.6 points and practice by 28.1 points compared to the control group. The intervention was perceived as acceptable and appropriate by the study participants and key stakeholders. Perceived barriers included reaching private network users and poor network in geographically remote areas, while enabling factors included mobile phone penetration, low cost, and shared responsibility.

**Conclusions:**

Mobile SMS is an effective, acceptable and appropriate health intervention to improve dengue prevention practices in communities. This intervention can be adopted as a promising tool for health education against dengue and other diseases.

## Background

Dengue is a fast emerging pandemic-prone viral disease identified as a major public health concern globally [1]. It is a rapidly emerging public health threat in Nepal, where the earliest cases were reported in 2005 with sporadic cases continuing with occasional major outbreaks [2]. Dengue is expanding throughout the districts of the southern belt and even up to the central mountains, where *Aedes spp.* vectors were recently found in the entomological surveillance [2–4]. A total of 917 confirmed dengue cases along with five deaths were reported in 2010 alone [2]. From 30 different districts within Nepal in 2016, the number of cases increased to 1529 along with one death reported. The biggest outbreak was reported from Chitwan district with 687 cases followed by Jhapa district with 405 cases [5]. The country-wide distribution of dengue fever cases was highly clustered around several districts, especially Chitwan and Jhapa, and showed high inter-annual and seasonal variation [6].

In line with the integrated vector management strategies developed by the World Health Organization (WHO), the major activities conducted in Nepal to control dengue vectors are search and destroy campaign, residual spraying, stakeholders orientation on vector control, and development of health education messages and dissemination through various media [2]. The standard messages developed for dengue awareness are delivered through leaflets, pamphlets, wall paints, street drama, FM radios and local newspapers. However, all these vector control measures are exclusively conducted as part of emergency responses to outbreaks [7]. Frequent dengue outbreaks in the past few years, with the geographical expansion of dengue vectors, and minimum vector control activities in Nepal suggest that knowledge, attitude, and practice (KAP) of dengue transmission, prevention, and vector control among the people are poor and require attention [8]. These trends indicate the urgent need to find effective interventions targetted to behavior change of individuals.

Health promotion for behavior change is always challenging and demands innovative solutions. Delivering health messages through short message service (SMS) based on mobile phones has been found to be beneficial for public health-related uses [9]. SMS gives an exciting opportunity for health promoters to engage personally with a large group of people irrespective of the model of their cellphones and at a relatively low cost [10]. Mobile text messaging is not only feasible and acceptable in the health care delivery platform, but the recipients generally perceive the health promotion intervention as useful and have also demonstrated a positive attitude in an interventional study of weight reduction in the United States [11]. Recent evidence has shown the potential use of mobile SMS to increase the health literacy of communities, making the intervention considered more cost-effective in contrast to traditional health education methods [12]. Mobile SMS was also found to be more effective compared to pamphlets to improve the KAP of mothers of preschool children [13]. Repeated health information exposure through mobile SMS encouraged people in dengue-endemic areas of Peru to improve their prevention practice against the vector borne disease [14].

In recent years, mobile phone penetration has been so extensive and rapid that almost all households in developing countries have adopted the technology. Mobile phone service can potentially facilitate the diffusing of health knowledge and good practices by reducing transaction costs, providing instant access to information to a large population and thereby improve the delivery of public services [15, 16]. In Nepal, the national census conducted in 2012 showed a massive increase in ownership of mobile phones among Nepalese people compared to previous years. Approximately 65% of households have mobile phones nationally and around 80% in Chitwan district, where the study was conducted [17]. New statistics reported by a national newspaper mentioned that mobile subscriptions have outnumbered the total population of the country (27.85 million subscriptions in 26.49 million population) [18].

These facts provide an excellent opportunity to introduce mobile-based health education as part of dengue control interventions. However, there is still no study which explores the effectiveness of this SMS intervention in the Nepalese setting. The aim of this study was to explore the effectiveness, acceptability, and appropriateness of the mobile SMS intervention in improving dengue preventive behavior in dengue endemic areas of Nepal.

## Methods

### Study design

This study was an implementation research that used sequential explanatory mixed-method design with intervention. The interventions were conducted using a non-randomized quasi-experimental design with three groups: one control group, one dengue prevention leaflet (DPL) only intervention group and one DPL with mobile SMS intervention group (DPL + SMS).

We measured the change in dengue preventive knowledge and practice of respondents through pre and post-intervention surveys. We also assessed the perceptions of community people towards acceptability and appropriateness of the interventions in the survey. Similarly, we interviewed key informants from stakeholder organizations to assess their perceptions of appropriateness, barriers, and enablers for the adoption of the mobile SMS intervention.

### Study setting

**Fig. 1:**
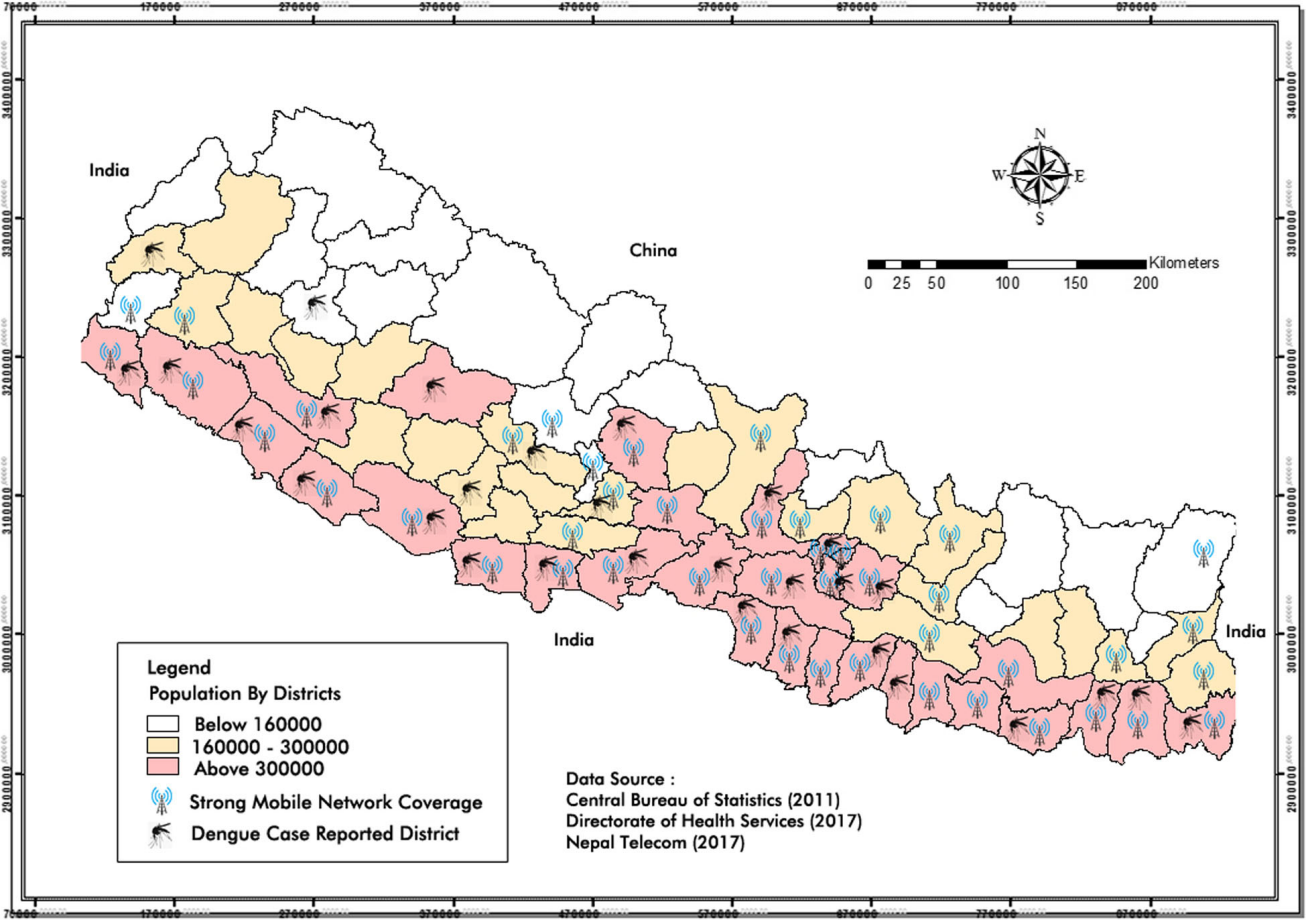
Research settings

Dengue fever is rapidly expanding its geographical range in all three ecological regions of Nepal [8]. The majority of the cases are being reported from the lowland districts (below 1500 m from sea level) which are densely populated and going through rapid urbanization [4, 5, 8]. The population of districts, dengue reported districts and districts with strong mobile network coverage are presented in Fig. 1. We opted for mobile SMS as the selected media to disseminate preventive health messages in this study keeping in mind the fact that the penetration of the Internet in Nepal is just 19.7% while 64.6% of households have mobile phones [19].

The dengue control program in Nepal is led by the Epidemiology and Disease Control Division (EDCD) and the activities are conducted at the district level by District (Public) Health Offices. Vector Control Inspectors (VCI) work as a focal point at the district level and coordinate with health workers and Female Community Health Volunteers (FCHV) from the peripheral level to implement the planned activities.

### Study site

The research was done in Ratnanagar Municipality (Ward Number 2) of Chitwan district in Nepal. Ward number 2 consists of 1063 households. We selected three study clusters in the distant corners of the ward to avoid cross-contamination. Chitwan was the highest hit area for dengue outbreaks in 2010, 2013 and 2016. The total population of Chitwan district is 579,984 [17]. The district has a tropical and sub-tropical climate in its lowlands and mid-mountain region, respectively. The study site lies in the lowlands region. Along with the climatic condition, the rapid urbanization of Ratnanagar Municipality facilitates the increasing wide-spread transmission of the dengue virus. The field activities of the study were conducted between September to December 2017.

### Research subjects

The FCHV Program of Nepal is recognized internationally as an exemplary volunteer Community Health Workers (CHW) program. One FCHV serves approximately 50 households in Nepal [20]. Ward number 2 of Ratnanagar Municipality has 1063 households. We considered one FCHV serving 50 households as a unit. We identified three distant corners of the ward for interventions and control to avoid contamination among study groups. We applied a lottery method to assign study groups. In each of the study groups, we purposively selected two FCHVs who were serving 100 adjacent households. We recruited the head of household or spouse as study participants from each household. To ensure the head of household or spouse in all three groups were comparable, we applied the same inclusion and exclusion criteria. A head of household or spouse was considered as eligible to participate if s/he has a cell phone which can receive and send SMS messages and s/he is literate enough to read a simple text message. We recruited 300 participants, involving the head of the households or spouse as a reasonable sample size in order to detect the power of more than 80% and expecting a mean difference above five points in knowledge between groups.

We used survey interview to assess the community’s perceived appropriateness and the acceptability of the mobile SMS health promotion about dengue disease. We further explored the appropriateness and acceptability using in-depth interviews with ten SMS recipients.

Representing different organizations and levels that could share the goal of health promotion envisioned by this study, three key stakeholders, i.e. Dengue Focal Point from EDCD, Public Health Officer from NHEICC, and Focal Points from NTC participated in the qualitative strand of this study. Enablers and barriers to the adoption of mobile SMS intervention were explored through interviewing of key stakeholders.

### Sampling technique

A total sampling of household heads or spouses in the study groups was done for the pre and post-intervention surveys. All household heads or spouses from the DPL + SMS intervention group were surveyed and interviewed for assessing the acceptability and appropriateness of the SMS intervention. The purposive sampling method was utilized to recruit key stakeholders from related organizations to assess stakeholders’ perceptions of the appropriateness of intervention and for exploring the potential enablers and barriers that might influence the adoption of intervention. We recruited these informants because they are the responsible representatives from the stakeholder organizations which share the same goal of disseminating dengue-related education to the community.

### Interventions

The interventions in this study included: a) dengue prevention leaflets delivered to each household by FCHV during home visits (DPL Only), b) dengue prevention leaflets combined with SMS reminder via mobile phone (DPL + SMS). Dengue prevention leaflets are the pamphlets developed by EDCD/NHEICC focusing on dengue prevention activities. After completion of the pre-intervention survey, FCHVs were mobilized to distribute the leaflets to each household of DPL only and DPL + SMS groups during their regular home visits. The SMS reminder consisted of dengue prevention related information transmitted through mobile phone text messages twice a week right after the pre-intervention survey. In 6 weeks, each of the study participants of the DPL + SMS group received a total of 24 SMSs. Two messages were sent in a day (Tuesday and Saturday) during dusk and dawn through a professional bulk SMS service provider. Every effort was made to ensure that the participants knew how to receive and read the message and the households did not incur any cost for receiving the messages. Efforts made were ensuring the literacy status of the study participants, the study participants owning the mobile that could receive SMS with scripts in Nepali language, and the SMS service provider not charging any cost for receiving the SMS.

### Dengue SMS development

The principal investigator and co-investigator first drafted the text messages with a reference from a published article [14]. These messages targeted three different goals: i) reminder of the dengue prevention leaflets, ii) search and destroy mosquito breeding places, i.e. water storage, garbage disposal, and water-holding solid waste in and around the houses, and iii) preventing exposure to bites of *Aedes* mosquitoes i.e., use of long sleeve clothes, insect repellents, bed nets, screening of windows and doors.

A participatory workshop was then conducted in EDCD to further discuss the text messages. The participants of the workshop were the focal persons from EDCD, health communication specialists from NHEICC, focal persons from NTC, and the representatives from the target population. The workshop ensured the relevance of the information, better wording, and comprehensiveness of the messages.

Before the intervention trial, the prepared messages were pilot tested in the similar setting for their readability in 10% of the sample population (i.e. ten people) having similar characteristics of the study site and the content was then finalized.

### Research instruments

This study used five different research instruments for quantitative and qualitative data collection (Table 1). The research instruments are available as supplementary file (Additional file 1) with this manuscript. The Knowledge-Practice questionnaire survey on dengue prevention was adapted from published research [8]. We assessed the reliability coefficient to measure the internal consistency of the questionnaire using Cronbach’s Alpha and obtained 0.8 and 0.7 for the knowledge and practice components, respectively. A structured questionnaire was used to assess the perception of participants who received the mobile SMS reminder. Acceptability of the technology and message content was assessed using a 13-item Likert-type scale ranging from 1 (Least Favorable) to 5 (Most Favorable) [11]. Appropriateness was assessed using a 5-item Likert-type scale ranging from 1 (Extremely inappropriate) to 7 (Extremely appropriate) [21, 22]. The Cronbach Alpha coefficients for acceptability and appropriateness scale were assessed as 0.75 and 0.71, respectively.

**Table 1:** Research tools

Research instruments	Respondents
Quantitative Tools	
Knowledge-Practice Questionnaire Survey	Head of households or spouse
Mobile SMS Acceptability Survey	Head of households or spouse
Mobile SMS Appropriateness Survey	Head of households or spouse
Qualitative Tools	
Participant’s Perspectives towards acceptability of Mobile SMS intervention	Head of households or spouse
Stakeholders’ Perspectives towards appropriateness of Mobile SMS intervention	Key stakeholders

We used a semi-structured questionnaire to assess the participants’ perspectives towards the acceptability of mobile SMS intervention. Similarly, a semi-structured questionnaire was used to assess the organizational stakeholders’ perspectives towards the appropriateness of the mobile SMS reminder. Specific questionnaires were used for interviewing the key stakeholders of all organizations involved.

Before using in the main study, the survey questionnaires were pre-tested among the members of community in Ratnanagar Municipality to check the flow, consistency and validity. The questionnaires filled in during pre-test survey were not included in the final analysis.

Six research assistants with an undergraduate degree in Public Health and experience in data collection were recruited and trained to assist the principal investigator during the data collection process. They collected quantitative data during the pre-test and post-test surveys.

### Data analysis

We used Epi Data 3.1 software for data entry and management and STATA 13.1 software for data analysis of quantitative data.

Descriptive analysis was done for the socio-demographic characteristics of the research participants. Chi-square test was used to compare these characteristics between the study groups. Mean scores with standard deviation (SD) for knowledge and practice before and after the intervention were calculated for all study groups. We used one-way ANOVA test to compare the knowledge and practice between study groups before implementation of the intervention. Similarly, we used paired t-tests to compare the change in knowledge and practice within groups and one-way ANOVA to compare the change observed between groups after the implementation of the intervention. We conducted multiple linear regression to evaluate the effectiveness of interventions controlling the potential confounding variables. All *p*-values were considered statistically significant at *p* < 0.05. Participants’ perspectives regarding acceptability and appropriateness of the intervention were assessed calculating the average agreement score based on the individual scores obtained in the Likert scale.

An interview guide was developed for the interviews, which were conducted in Nepali language and tape recorded, then manually transcribed into English language. Open Code 4.03 Software was used for coding and thematic analysis of the qualitative data. Determination of appropriateness of the intervention was done through thematic analysis of the qualitative findings of the research. The thematic analyses of the acceptability of the technology and message content were used to support the evidence generated through quantitative technique. Enablers and barriers towards the adoption of the intervention were determined and presented based on the reporting done by the stakeholders during key informant interviews.

## Results

### Sociodemographic characteristics of study population

All of the heads of household or spouses who were approached participated in the study. Most participants from the study were female (57%), had attained education higher than secondary level (40%), and belonged to the Brahmin/Chhetri ethnic group (48%). The mean age of the respondents was 40 ± 11 SD years, with the majority (65%) in the age group 30–49 years old. Only a little more than half (53%) of respondents reported their annual household income to be more than NPR 250,000. Among the sociodemographic characteristics, sex (*p* = 0.000), age (*p* = 0.005), and ethnicity (*p* = 0.000) were significantly different between the control, DPL and DPL + SMS groups (Table 2).

**Table 2:** Sociodemographic characteristics of participants

Variables	Total n (%)	Control n (%)	DPL^#^ Only n (%)	DPL + SMS n (%)	*p*-value
Total participants	300 (100)	100 (100)	100 (100)	100 (100)	
Sex					0.000
Male	130 (43)	64 (64)	31 (31)	35 (35)	
Female	170 (57)	36 (36)	69 (69)	65 (65)	
Age group in years					0.005
< 30	52 (17)	17 (17)	23 (23)	12 (12)	
30–49	195 (65)	58 (58)	70 (70)	67 (67)	
> 49	53 (18)	25 (25)	7 (7)	21 (21)	
Education level					0.330
Literate	37 (12)	16 (16)	8 (8)	13 (13)	
Primary	46 (15)	13 (13)	17 (17)	16 (16)	
Secondary	97 (33)	29 (29)	40 (40)	28 (28)	
Higher than secondary	120 (40)	42 (42)	35 (35)	43 (43)	
Ethnicity					0.000
Brahmin/Chhetri	143 (48)	35 (35)	38 (38)	70 (70)	
Janajati	109 (36)	46 (46)	37 (37)	26 (26)	
Others*	48 (16)	19 (19)	25 (25)	4 (4)	
Annual household income**					0.273
< 250,000	140 (47)	53 (53)	42 (42)	45 (45)	
> 250,000	160 (53)	47 (47)	58 (58)	55 (55)	

All *p*-values are based on chi-square analysis of numbers in the three study clusters; ^#^ DPL = Dengue Prevention Leaflet; ^a^ Others include Dalit, Terai/Madhesi and Muslim respondents; ^b^ In Nepalese currency (NPR), (1 USD = 107 NPR as of October 2017)

### Effects of intervention on knowledge and practice

The highest mean difference in overall knowledge score when compared between study groups was seen in the DPL + SMS group with 32.7 ± 13.7 SD and followed by a less than half score of 13.3 ± 8.8 in the DPL only group. The difference was statistically significant (*p* = 0.000) (Table 3). The mean difference in knowledge was statistically different between groups before the intervention. Similarly, there was a stark difference between the mean difference in overall practice score of the DPL + SMS group (27.9 ± 11.4 SD) compared to the DPL only group (4.9 ± 5.4 SD). The difference was statistically very significant (*p* = 0.000) (Table 4). There was also a statistically significant difference in the pre-intervention overall practice between groups.

**Table 3:** Dengue preventive knowledge within and between groups

	Control	DPL^b^ Only	DPL + SMS	*p*-value^a^
Preintervention mean score	71.4 ± 12.2	68.3 ± 14.01	64.1 ± 13.7	0.0006
Postintervention mean score	71.7 ± 11.8	81.6 ± 10.9	96.8 ± 2.7	0.000
Mean score difference	0.2 ± 4.5	13.3 ± 8.8	32.7 ± 13.7	0.000

^a^One way ANOVA test; ^b^DPL = Dengue Prevention Leaflet

**Table 4:** Dengue preventive practice within and between groups

	Control	DPL^b^ Only	DPL + SMS	*p*-value^a^
Preintervention mean score	62.5 ± 9.2	60.2 ± 12.3	56.8 ± 12.2	0.0016
Postintervention mean score	63.5 ± 9.5	65.1 ± 11.9	84.7 ± 5.5	0.000
Mean score difference	1 ± 4.01	4.9 ± 5.4	27.9 ± 11.4	0.000

^a^One way ANOVA test; ^b^DPL = Dengue Prevention Leaflet

Multivariate analysis of practice difference between groups showed that the DPL + SMS intervention was found to be effective in improving the practice of respondents by 24.1 points compared to the control group with high significance. The results demonstrate that the DPL + SMS intervention brought change in practice better than the DPL only intervention (Table 5).

**Table 5:** Multivariate linear regression for denge preventive practice difference

Variable	Model I		Model II	
	B	CI 95%	B	CI 95%
Study group				
Control (Ref)				
DPL^a^ Only	3.4*	1.6–5.2	3.1*	1.3–4.8
DPL + SMS	24.3*	22.3–26.2	24.1*	22.2–25.9
Preintervention practice score	−0.4*	−0.4 – −0.3	−0.39*	−0.4 – −0.3
Sex				
Male (Ref)				
Female	−0.6	−2.2 – 0.9		
Age group in years				
< 30 (Ref)				
30–49	−0.5	− 2.5 – 1.5		
> 49	0.3	−2.3 – 2.9		
Ethnicity				
Brahmin/Chhetri (Ref)				
Janajati	−1.1	−2.8 – 0.5	−0.9	− 2.6 – 0.6
Others	−2.9*	−5.1 – −0.7	−2.8*	−4.9 – − 0.6
R^2^	0.8086		0.8075	

*p-value< 0.05; *CI* Confidence Interval, ^a^ *DPL* Dengue Prevention Leaflet, *Ref* Reference

### Acceptability of mobile SMS intervention

All respondents considered mobile SMS as a highly acceptable media for disseminating health promotion information to them with the average mean score on usefulness and attitudes of SMS of 4.4 on the 5-point scale assessment. The respondents perceived that receiving mobile messages for dengue prevention was enjoyable, informative, and trustworthy. They agreed that the content of the message could be used as a reference for dengue prevention practices. They even said that receiving mobile messages in this regard was not irritating. However, one respondent mentioned that frequently receiving message could be irritating at times.

*“Once I felt irritated but I think one message per day is good for the information.”* (Participant 4)

It was interesting to know from almost every interviewee that they would recommend for all people to receive this kind of health message to help them practice dengue preventive behavior.

In addition to this positive feedback, the information on process measures of the mobile SMS was also assessed during the survey as an intention to receive SMS, the timing of message reading and extent of message reading. Three fourths (75%) of the respondents reported that they would like to receive only one message in a day while 25% of them showed a willingness to receive up to two messages in a day. Almost all of the respondents (96%) read the messages right away. Only 4% of them read the messages when there was enough time. More than half of the respondents (66%) read the whole message when they received it, while 32% of them mentioned that they read about three-quarters of the message.

The findings were similar when the respondents were interviewed for exploring their experiences and perceptions of receiving mobile SMS related to dengue prevention. Eight major themes emerged from the interviews including cue to action, informative, useful, entertainment value, support, intention to receive, timing of reading message, and sharing of message.

### Appropriateness of mobile SMS intervention

#### Community perceptions

When we assessed the recipients’ perceptions towards the mobile SMS intervention in this study, the average score for the overall appropriateness of the intervention was 5.8, close to very appropriate in the 7-point scale assessment. More than half (64%) of the respondents scored as very appropriate (6 on the 7-point scale assessment) when they were asked if mobile SMS is the best choice for disseminating health education among other possible alternatives. Similarly, 55% of the respondents perceived that combining dengue prevention leaflet with mobile SMS for giving dengue prevention awareness messages was very appropriate. A slightly higher number of respondents (58%) considered mobile SMS alone as only somewhat appropriate (5 on the 7-point scale) to change the behavior of people if not combined with dengue prevention leaflet.

#### Stakeholders’ perception

The major themes that emerged from interviewing key stakeholders were mobile phone penetration in the community, existing practices, organizational readiness, partnerships among stakeholders, and conventional methods versus mobile technology. These themes reflect stakeholders’ perceptions that pointed towards the possibility of adopting mobile SMS as a useful and appropriate health awareness disseminating tool at the present time and setting.

*“ … … . majority people have mobile phones in their hands in Nepal … … …… .. Since mobile phones are extensively used, it won’t be difficult to send via SMS. It can go smoothly … … .”Male, Engineer, NTC*

*“ … … We don’t have specifically in disease control yet, but for health promotion, like in special events, risk factors of NCDs, and we have another called as MY Year Campaign targeting NCDs. We have just started doing SMS messages for this and nutrition. … ..” Male, Public Health Officer, NHEICC*

All of the stakeholders mentioned that their organizations are capacitated in terms of finance, technology, and infrastructure to adopt mobile SMS as a viable health awareness intervention.

*“ … … The main strategy to control dengue is through search and destroy, through programs to increase public awareness. This is done in all districts. We have been mobilizing various media, uploading awareness information in the website and can readily adopt mobile SMS as a tool too. So, our division very much ready for this … …… ” Male, VCI, EDCD*

The informants considered the present time appropriate to adopt modern technology such as mobile applications and SMS for message dissemination instead of with the conventional methods. It was explained that people are more accustomed to using mobile phones in their daily lives, so it is appropriate and realistic to shift towards mobile SMS for health promotion.

*“ … ..more than 90% people are busy in their phones, they have a habit of using phones, SMS, I would like to inform you that this is very realistic and appropriate … ..” Male, VCI, EDCD*

*“ … … . And it won’t be very costly … . We had our investment in conventional methods like print media and others, we just have to shift from that to this. So, I feel if we have willingness there won’t be any financial issues … .” Male, PHO, NHEICC*

### Barriers and enablers influencing mobile SMS intervention

Four major themes emerged when we interviewed the key informants during this study for exploring the enablers to use mobile SMS intervention for dengue-related awareness. They are mobile phone penetration, low financial cost, shared responsibility and corporate social responsibility.

*“ … .mobile SMS is very appropriate. As I said earlier … …… … mobile phones are very common, people are user-friendly to it … … ” Male, VCI, EDCD*

*“ … … The cost of SMS is very low. That is also no difficult … ..” Male, Engineer, NTC*

Similarly, when we processed the information they shared during the interviews, three themes emerged as barriers to mobile SMS intervention: the purpose of using mobile phones, reaching private network users, and the poor network in geographically remote areas.

*“ … .. We are talking about sending health-relatedmessages. It depends on the target population if they are using network/phone just for making calls or to receive or send messages too … .” Male, Engineer, NTC*

## Discussion

This implementation research presents evidence that conventional health education methods when combined with mobile SMS can bring major improvement in the knowledge and practice of community people towards dengue prevention in Nepal. With this purpose, this study is the first of its kind in Nepal to compare the difference in effectiveness of conventional health promotion media (pamphlets and leaflets) alone and interventions combining those with mobile SMS on knowledge and practice towards dengue prevention. To our knowledge, this study is a pioneer in establishing concrete evidence that mobile SMS is an acceptable and appropriate contemporary media to deliver dengue preventive health messages to the general public. To our knowledge, this study is the first in presenting the perceptions of people towards the appropriateness of mobile SMS interventions in delivering dengue preventive health messages.

The findings of this research should be interpreted in light of the study’s limitations. This study was conducted in a specific residential area of Chitwan district in Nepal. Therefore, generalizability is limited to similar sites and households. However, this intervention can be replicated in countries and settings with high mobile penetration but low Internet access. This study assessed dengue preventive practices through a survey where only data on reported practices were collected, and no actual practices of the respondents were observed. Additionally, we assessed the immediate difference seen in knowledge and practice of participant right after the exposure to the intervention. Further research over an extended period of time and in diverse settings is necessary to understand the long-term influence of the intervention implemented in this study.

### Effectiveness of mobile SMS intervention

The research findings showed that the health awareness interventions adopted in this study, i.e. dengue prevention leaflet only and combining dengue prevention leaflet with mobile SMS are both effective in improving knowledge and practice of people concerning dengue prevention. However, when the intervention was combined with mobile SMS, the knowledge difference was increased by more than two-fold, and practice difference was improved by more than five-fold compared to the group where only dengue prevention leaflet was delivered. This finding may be because people are more accepting of the information through mobile SMS and might have read the information repeatedly. This result is closely similar to a study in China that explored different communication interventions to improve rabies-related KAP and reported that combining SMS and regular information sessions can produce better effects [23]. The result is also in accordance with a field experiment conducted in Peru which reported that positive changes were seen in household behavior when exposure of preventive health information related to dengue was given through mobile phones in order to study participants’ responses and reactions [14].

### Acceptability and appropriateness of mobile SMS intervention

This study reveals that the respondents perceived mobile SMS as a highly acceptable media for receiving dengue preventive messages to improve their existing knowledge and practice. The findings of this study are consistent with the findings of a similar study about mobile health intervention to sustain weight loss which reported that SMS is a feasible, acceptable and useful mode for weight loss interventions [11]. The results were also similar to some literature reviews which presented the promising feasibility and acceptability of mobile SMS in improving preventive health behavior of people [24, 25].

This study further demonstrated that people in the affected communities considered mobile SMS as the appropriate mode of disseminating dengue preventive messages. Interestingly, almost equal number of respondents reported that they find SMS alone and SMS combined with dengue prevention leaflet as appropriate intervention to enhance their knowledge and practices related to dengue prevention. When considering terminology adopted by Proctor and his team, appropriateness is conceptually similar to acceptability and also related to feasibility in terms of constructs [26]. In this light, we can claim that our findings concerning the appropriateness of mobile SMS interventions in this study are similar to the findings in all of the literature mentioned above which reported mobile SMS intervention as an acceptable and feasible mode of health promotion and intervention [11, 24, 25]. Furthermore, the key stakeholders responsible for disseminating dengue preventive awareness messages in Nepal claimed that mobile SMS is an appropriate media in the present context to enhance the health knowledge and practice of community people. Therefore, mobile SMS is the most acceptable and appropriate media to reach the large section of the population in Nepal who are at risk of dengue disease at present because this media is the most effective and convenient way of reaching the target population without cellular data or Wi-Fi signals (Fig. 1). However, we experienced no interaction from message recipients through mobile SMS as this option was not made available in this intervention. We can potentially overcome this limitation using interactive SMS application or possibly use social media networks in the communities where the Internet coverage is good [27].

### Barriers and enablers to mobile SMS intervention

The primary barrier was a poor network in several geographically remote areas, especially in the hills and mountains of Nepal. Although the authorities from telecommunication providers claim that they are scaling up the service throughout the country and improving the quality of service, there is limited access or fluctuations of the network in some residential areas of hills and mountains. This finding substantiates the results of earlier conclusions where limited network coverage and fluctuating network are reported as barriers [28]. The purpose of using mobile phone by community people is perceived as another barrier in this study which is similar to the finding of an experimental study done in Myanmar where they considered lack of community familiarity with text messages as a potential barrier [28].

Reaching private network users is also perceived as a barrier in our study. Since there are several private mobile service networks providing services to the people, connecting with the user groups in those networks could be a challenge. However, an effective and planned coordination with those network providers could be the enabler to reach the section of people who are using their networks for communication.

Mobile phone penetration is perceived as the important enabler by the stakeholders in the context of implementing this intervention. In the present context where mobile phones have become an integral part of people’s lives, this available option for intervention can be a widely accepted medium of health message delivery to a large audience all at once.

Low financial cost for the intervention emerged as another enabler in our study which is reported by a similar study in Peru claiming text messages can not only be used in dengue prevention but has the potential for use in other disease prevention programs to identify risk behaviors and remedies [14]. Another study conducted on mobile phone text messaging intervention for cervical cancer screening also reported the SMS intervention is a low-cost and effective method to reach at-risk populations [29].

We identified shared responsibility and corporate social responsibility as another enabler in our study which is commonly practiced in Nepal as an approach involving multi-sectoral coordination. This charitable community action helps in minimizing the cost burden of health promotion in a specific division and imparts a sense of health-related responsibility in other non-health sectors as well.

The results of this study indicate that it is the appropriate time to consider mobile SMS as a crucial public health advocacy tool to enhance preventive health behavior of people. However, this intervention must be done through effective coordination and collaboration among the stakeholders while exploring the new and more interactive text messaging technology. There is still much room for further exploration about the long-term effects and benefits of the intervention.

## Conclusions

This study demonstrated that combining mobile SMS with conventional methods such as dengue prevention leaflets can produce a better change in knowledge and practice of people than conventional methods alone. Mobile SMS is effective, acceptable and appropriate intervention to improve the dengue preventive knowledge and behavior of people. The results provide evidence concerning the potential importance and usefulness of combining mobile SMS with dengue preventive leaflets as an effective health promotion intervention for dengue prevention; an emerging and promising approach for health education for dengue, other diseases, and risk factors. We recommend for the adoption of mobile SMS as the complimentary channel of communication to disseminate health preventive messages to community people through effective coordination and collaboration with stakeholders.

Communication modes such as mobile SMS can serve as effective, acceptable and appropriate media to disseminate health promotion and disease prevention messages to a large group of people considering its penetration in the community.

## Supplementary information

**Additional file 1.** Research tools, Survey instruments (DOCX 43 kb)

## Data Availability

The data and materials used in this study will be made available from the corresponding author upon reasonable request. However, the required information related to the research is already presented in this manuscript.

## Abbreviations

CBS: Central Bureau of Statistics
CHW: Community Health Worker
DENV: Dengue Virus
DF: Dengue Fever
DoHS: Department of Health Services
DPL: Dengue Prevention Leaflets
EDCD: Epidemiology and Disease Control Division
FCHV: Female Community Health Volunteer
IDI: In-depth Interview
IEC: Information, Education and Communication
IVM: Integrated Vector Management
KAP: Knowledge Attitude Practice
KII: Key Informant Interview
NHEICC: National Health Education Information and Communication Centre
NHRC: Nepal Health Research Council
NTC: Nepal Tele Communication
NVBDCP: National Vector Borne Disease Control Programme
PRISM: Practical, Robust Implementation and Sustainability Model
SMS: Short Message Service
TAM: Technology Acceptance Model
UGM: Universitas Gadjah Mada
WHO: World Health Organization
