# Supplementary material for: The addition of mobile SMS effectively improves dengue prevention practices in community: an implementation study in Nepal
Source: BMC Health Serv Res. 2019 Oct 15;19:699. doi: 10.1186/s12913-019-4541-z (PMC6794782; doi:10.1186/s12913-019-4541-z)
Supplement: Supplementary file 1 — Additional file 1. Research tools, Survey instruments (DOCX 43 kb) [file 12913_2019_4541_MOESM1_ESM.docx]

**Knowledge and Behaviour Assessment Survey**

*Adapted from Dhimal, M. et al., 2014*

**“Acceptability, appropriateness and effectiveness of mobile SMS intervention to enhance knowledge and behaviors of community people towards dengue prevention in Nepal”**

**Objectives**: The objective of this assessment is to assess the level of knowledge related to dengue prevention practices among the research participants living in selected clusters of Ratnanagar Municipality, Chitwan, Nepal.

**Instruction:** This questionnaire should be filled in for the head of the household or the spouse considering the exclusion and inclusion criteria mentioned in the study. The assessment should be made in for every household during pre-test and post-test survey in all three clusters. Tick the correct answer (Yes/No/DO Not Know) and fill in the details as appropriate. The answers obtained will be used only for the study purpose and will be kept confidential.

**Researcher: Ashmin Hari Bhattarai**

| Name of Cluster: | A ……. B …… C ……. |
| --- | --- |
| Code Number | ………………………………… |
| Position in the Family | Head of the family …….  Spouse …… |
| Name of Interviewer: | …. ………………………………. |
| Survey | Pre-test Survey …..  Post-test Survey….. |

1. **Demographic characteristics of respondent**

| S. N. | Age | ……. (in completed years) |
| --- | --- | --- |
| 1 | Sex | Male ….  Female ….  Third Gender …. |
| 2 | Education | Read/write ….  Primary ….  Secondary ….  Higher Secondary or above …. |
| 3 | Ethnicity | Brahmin/Chhetri ….  Tarai/Madhesi ….  Dalits ….  Newar ….  Janajati ….  Muslim ….  Other …. |
| 4 | Annual Household Income | ……………….. (in NPR) |

1. **Knowledge of Dengue Prevention**

| **S.N.** | **Variables** | **Yes** | **No** | **Do not know** |
| --- | --- | --- | --- | --- |
|  | Have you heard about dengue?  (Continue the interview if s/he has heard about it) |  |  | **NA** |
|  |  |  |  |  |
|  | **Knowledge of transmission** |  |  |  |
| **1** | Can all mosquitoes transmit dengue fever? |  |  |  |
| **2** | Do the *Aedes* mosquitoes transmit dengue fever? |  |  |  |
| **3** | Do flies transmit Dengue fever? |  |  |  |
| **4** | Do ticks transmit Dengue fever? |  |  |  |
| **5** | Does ordinary person to person contact transmit Dengue fever? |  |  |  |
| **6** | Is Dengue fever transmitted through food and water? |  |  |  |
| **7** | Can dengue fever be transmitted by blood transfusion? |  |  |  |
| **8** | When are the Dengue mosquitoes likely to feed/bite? |  |  |  |
|  | Night time |  |  |  |
|  | Day time |  |  |  |
|  | Both day and night |  |  |  |
|  |  |  |  |  |
|  | **Knowledge on preventing exposure to bites of *Aedes* mosquitoes (Mosquito-man contact)** |  |  |  |
| **1** | Can covering body with clothes prevent mosquito bites? |  |  |  |
| **2** | Can Mosquito repellents/cream prevent mosquito bites? |  |  |  |
| **3** | Can use of fan prevent mosquito bites? |  |  |  |
| **4** | Can use of mosquito coil reduce mosquitoes? |  |  |  |
| **5** | Can use of windows screen reduce mosquitoes? |  |  |  |
| **6** | Can use of bed nets while sleeping prevent mosquito bites? |  |  |  |
| **7** | Can use of smoke to drive away mosquitoes reduce mosquitoes? |  |  |  |
|  |  |  |  |  |
|  | **Knowledge on preventing production of adult mosquitoes (Search and destroy)** |  |  |  |
| **8** | Can covering water containers in the home prevent mosquito breeding? |  |  |  |
| **9** | Can frequent/routine cleaning of water containers and ditches around the houses prevent mosquito breeding? |  |  |  |
| **10** | Can turning containers upside down to avoid water collection reduce mosquitoes? |  |  |  |
| **11** | Can eliminating standing water around the houses reduce mosquitoes? |  |  |  |
| **12** | Can disposing water holding containers such as tires, parts of autmobiles, plastic bottles, crack pots, etc. reduces mosquitoes? |  |  |  |
| **13** | Can cleaning garbage/trash reduce mosquitoes? |  |  |  |
| **14** | Can cutting down bushes in the yards reduce mosquitoes? |  |  |  |
| **15** | Can using mosquito eating fish reduce mosquitoes? |  |  |  |
| **16** | Can using insecticide sprays reduce mosquitoes? |  |  |  |
| **17** | Can using professional pest controls reduce mosquitoes? |  |  |  |

1. **Which prevention approach against dengue fever do you practice?**

| **S.N.** | **Variables** | **Yes** | **No** |
| --- | --- | --- | --- |
|  | **Preventing exposure to bites of *Aedes* mosquitoes (Mosquito-man contact)** |  |  |
| **1** | Covering body with clothes |  |  |
| **2** | Use mosquito repellents/cream |  |  |
| **3** | Use of fan |  |  |
| **4** | Use mosquito coil to reduce mosquitoes |  |  |
| **5** | Use windows screen to reduce mosquitoes |  |  |
| **6** | Use of bed nets while sleeping |  |  |
| **7** | Use of smoke to drive away mosquitoes |  |  |
|  |  |  |  |
|  | **Preventing production of adult mosquitoes (Search and destroy)** |  |  |
| **8** | Cover water containers in the home |  |  |
| **9** | Frequent/routine cleaning of water containers and ditches around the houses |  |  |
| **10** | Turning containers upside down to avoid water collection |  |  |
| **11** | Eliminate standing water around the houses to reduce mosquitoes |  |  |
| **12** | Disposing water holding containers such as tires, parts of autmobiles, plastic bottles, crack pots, etc. |  |  |
| **13** | Cleaning garbage/trash |  |  |
| **14** | Cut down bushes in the yards to reduce mosquitoes |  |  |
| **15** | Use mosquito eating fish to reduce mosquitoes |  |  |
| **16** | Use insecticide sprays to reduce mosquitoes |  |  |
| **17** | Use professional pest controls to reduce mosquitoes |  |  |

**Contact person**

If you have any questions regarding this study, you may contact:

Principal Investigator: Ashmin Hari Bhattarai

E-mail: [ashmin.bhattarai@gmail.com](mailto:ashmin.bhattarai@gmail.com)

Phone Number: +977-9855041558 (Nepal) /+6282195049126 (Indonesia)

**Thank you!**

**Acceptability Questionnaire Survey for Intervention Recipients**

*Adapted from Shaw et al., 2013*

**“Acceptability, appropriateness and effectiveness of mobile SMS intervention to enhance knowledge and behaviors of community people towards dengue prevention in Nepal”**

**Directions:** This tool should be filled in for the participants who received the mobile SMS messages as intervention in this study. The assessment should be made for every participant after finishing of post-test survey in Cluster C.

**Objectives**: The objective of this assessment is to assess the perception of research participants (intervention recipients) regarding the acceptability of mobile SMS messages for dengue prevention.

**Describe the Reason for the interview:**

*We are here to learn about your experiences with receiving the mobile SMS for dengue prevention activities.*

*Would you be willing to answer some questions regarding your experiences?*

*Please understand that there is no compulsion for you and you are free to decide if you choose not to participate.*

**If no***: Thank them for their time*

**If yes***: Review the purpose of the study and the types of questions that are going to be asked. Remind participants that the interview will be audio-taped.*

**Researcher: Ashmin Hari Bhattarai**

| Code Number | ………………………………… |
| --- | --- |
| Name of Interviewer: | …….………………………………. |

**Attitudes and Behaviors Scale towards Dengue Prevention SMS**

1 = completely disagree, 3 = neutral (neither disagree nor agree), and 5 = completely agree

| **SN** | **Items** | **Score** |
| --- | --- | --- |
| 1 | I feel that receiving the text messages is enjoyable | 1 2 3 4 5 |
| 2 | I feel that receiving the text messages is pleasant | 1 2 3 4 5 |
| 3 | I feel that the text messages are a good source for timely information | 1 2 3 4 5 |
| 4 | The text messages provide the information I need for dengue vector control activities | 1 2 3 4 5 |
| 5 | I feel that the text messages are irritating | 1 2 3 4 5 |
| 6 | I feel that the text messages are too much | 1 2 3 4 5 |
| 7 | Content in the text messages are often annoying | 1 2 3 4 5 |
| 8 | I use the text messages as a reference for dengue vector control | 1 2 3 4 5 |
| 9 | I trust the text messages | 1 2 3 4 5 |
| 10 | Overall, I like the text messages | 1 2 3 4 5 |

11. I am willing to receive text messages for dengue vector control activities:

1. One message a day
2. Two messages a day
3. Three messages a day
4. Four messages a day
5. More than four messages a day

12. What do you do when you receive a text message about dengue vector control practices?

1. Ignore it completely
2. Read it occasionally
3. Read it after accumulating too many of them
4. Read it when I get time
5. Read it right away

13. How much do you read messages you received?

1. Not at all
2. Read about a quarter of a message
3. Read about half of a message
4. Read about three-quarters of a message
5. Read the whole message

**Closing**

*Thank you for participating in our research study. Please understand that your answers will remain confidential. Give them follow-up contact information such as the telephone number of the PI.*

**Contact person**

If you have any questions regarding this study, you may contact:

Principal Investigator: Ashmin Hari Bhattarai

E-mail: [ashmin.bhattarai@gmail.com](mailto:ashmin.bhattarai@gmail.com)

Phone Number: +977-9855041558 (Nepal) /+6282195049126 (Indonesia)

**Thank you!**

**In depth interview Guide for Acceptability of Technology and Message Content**

*Adapted from Shaw et al., 2013*

**“Acceptability, appropriateness and effectiveness of mobile SMS intervention to enhance knowledge and behaviors of community people towards dengue prevention in Nepal”**

**Directions:** This tool should be used for interviewing the participants who received the mobile SMS messages as intervention in this study. The interview should be conducted after finishing of post-test survey in Cluster C and continued until the data saturation is obtained.

**Objectives**: The objective of this assessment is to assess the perception of research participants (intervention recipients) regarding the acceptability of mobile SMS messages for dengue prevention.

**Describe the Reason for the interview:**

*We are here to learn about your experiences with receiving the mobile SMS for dengue prevention activities.*

*Would you be willing to answer some questions regarding your experiences?*

*Please understand that there is no compulsion for you and you are free to decide if you choose not to participate.*

**If no***: Thank them for their time*

**If yes***: Review the purpose of the study and the types of questions that are going to be asked. Remind participants that the interview will be audio-taped.*

**Researcher: Ashmin Hari Bhattarai**

| Code Number | ………………………………… |
| --- | --- |
| Name of Interviewer: | …..………………………………. |

**Questions:**

**Perceived Usefulness**

1. How useful did you find the text messages to be in helping you perform dengue vector control activities in and around your house?

*Probe:* What about search and destroy of mosquito breeding places? Avoiding contact with mosquitoes?

**Attitudes towards the receipt of intervention**

1. How do you feel about getting these messages for dengue vector control activities?
2. How much did you like the messages you read?
3. How did it make you feel to get these on your cell phone?
4. Did you have any problems with accessing or receiving the messages on your phone?
5. What worked for you?
6. What did not work for you?

**Adherence to the delivered intervention**

1. How often did you read the messages?
2. Did you read them multiple times?
3. Are there times when you did not read the messages?
4. How do you feel about how often (frequency) you got the messages?
5. Did you share the messages?

**Check for other ideas and suggestions overall**

1. Would you recommend getting these messages to someone else?
2. Do you have any other suggestions or thoughts?

**Field notes:**

Do not forget to record information describing the interview experience, any impressions or events that occurred during the interview.

**Closing**

*Thank you for participating in our research study. Please understand that your answers will remain confidential. Give them follow-up contact information such as the telephone number of the PI.*

**Contact person**

If you have any questions regarding this study, you may contact:

Principal Investigator: Ashmin Hari Bhattarai

E-mail: [ashmin.bhattarai@gmail.com](mailto:ashmin.bhattarai@gmail.com)

Phone Number: +977-9855041558 (Nepal) /+6282195049126 (Indonesia)

**Thank you!**

**Appropriateness Assessment Guide for Intervention Recipients**

*Adapted from Dharmar et al., 2007 and Thom et al., 2004*

**“Acceptability, appropriateness and effectiveness of mobile SMS intervention to enhance knowledge and behaviors of community people towards dengue prevention in Nepal”**

**Directions:** This tool should be filled in for the participants who received the mobile SMS messages as intervention in this study. The assessment should be made for every participant after finishing of post-test survey in Cluster C.

**Objectives**: The objective of this assessment is to assess the perception of research participants (intervention recipients) regarding the appropriateness of mobile SMS messages for dengue prevention.

**Researcher: Ashmin Hari Bhattarai**

| Code Number | ………………………………… |
| --- | --- |
| Name of Interviewer: | …………………………………… |

**Appropriateness Scale towards Dengue Vector Control SMS**

1 = extremely inappropriate, 2 = very inappropriate, 3 = somewhat inappropriate, 4 = intermediate, 5 = somewhat appropriate, 6 = very appropriate, and 7 = extremely appropriate

| **SN** | **Items** | **Score** |
| --- | --- | --- |
| 1 | Everything considered, can mobile SMS convey you the dengue preventive message as you intended to receive? | 1 2 3 4 5 6 7 |
| 2 | Considering pros and cons of mobile SMS, is this the best choice for disseminating health education among other possible alternatives? | 1 2 3 4 5 6 7 |
| 3 | Everything considered, how appropriate was mobile SMS for dengue vector control awareness to you? | 1 2 3 4 5 6 7 |
| 4 | How appropriate it was to combine dengue prevention leaflet with mobile SMS for giving dengue prevention awareness messages to you? | 1 2 3 4 5 6 7 |
| 5 | Would you consider mobile SMS alone as appropriate for willingness to change behavior if you were not given dengue prevention leaflets also? | 1 2 3 4 5 6 7 |

**Contact person**

If you have any questions regarding this study, you may contact:

Principal Investigator: Ashmin Hari Bhattarai

E-mail: [ashmin.bhattarai@gmail.com](mailto:ashmin.bhattarai@gmail.com)

Phone Number: +977-9855041558 (Nepal) /+6282195049126 (Indonesia)

**Thank you!**

**Key Informant Interview Guide**

**A) General**

Sex: ………………………………………………

Level of Education: ……………………………………….

Designation: …………………………………………….

Area of Expertise: …………………………………………..

Total Years of Service: ………………………………………

**B) Interview Questions for Focal Person at EDCD**

1. How do you consider about incorporating mobile SMS intervention in regular dengue awareness program? (*Probing: Do you think mobile SMS delivered to individuals will be effective in bringing positive change in behaviour of community people? Is there any previous experience?*)
2. To what extent do you think the goal of enhancing knowledge and preventive health behaviour of community people through mobile SMS intervention realistic and achievable?
3. Do you think your organization can adopt mobile SMS as health educational tool for community people with current organizational readiness? (Probing: *Talk about the technical, financial and infrastructural requirements and the current regulations*)
4. How do you see the possibility of coordinating with key stakeholders for implementing this intervention? (*Probing: discuss about the coordination with NHEICC and NTC*)
5. What could be the potential barriers for adopting mobile SMS intervention for health education to community people?
6. What could be the potential enablers for adopting mobile SMS intervention for health education to community people?
7. Is there anything else you would like to add or share that is relevant to his discussion?

**C) Interview Questions for Focal Person at NHEICC**

1. How do you consider about incorporating mobile SMS intervention in regular health awareness program? (*Probing: Do you think mobile SMS delivered to individuals will be effective in bringing positive change in behaviour of community people? Is there any previous experience?*)
2. To what extent do you think the goal of enhancing knowledge and preventive health behaviour of community people through mobile SMS intervention realistic and achievable?
3. Do you think your organization can adopt mobile SMS as health educational tool for community people with current organizational readiness? (Probing: *Talk about the technical, financial and infrastructural requirements and the current regulations*)
4. How do you see the possibility of coordinating with key stakeholders for implementing this intervention? (*Probing: give example of dengue awareness activities and possibility of coordination with EDCD and NTC*)
5. What could be the potential barriers for adopting mobile SMS intervention for health education to community people?
6. What could be the potential enablers for adopting mobile SMS intervention for health education to community people?
7. Is there anything else you would like to add or share that is relevant to his discussion?

**D) Interview Questions for Focal Person at NTC**

1. How do you consider about sending health messages to community people via mobile SMS messages? (*Probing: Discuss about mobile penetration in the community, coverage and strength of mobile networks*)
2. To what extent do you think the goal of enhancing knowledge and preventive health behaviour of community people through mobile SMS intervention realistic and achievable?
3. Do you think your organization can support in adopting mobile SMS as health educational tool for community people with current organizational readiness? (Probing: *Talk about the technical, financial and infrastructural requirements and the current regulations*)
4. How do you see the possibility of coordinating with key stakeholders for implementing this intervention? (*Probing: give example of dengue awareness activities and possibility of coordination with EDCD and NHEICC, the corporate social responsibility of NTC, etc.*)
5. What could be the potential barriers for adopting mobile SMS intervention for health education to community people?
6. What could be the potential enablers for adopting mobile SMS intervention for health education to community people?
7. Is there anything else you would like to add or share that is relevant to his discussion?

**Thank you!**

**Mobile SMS Readability Questionnaire Survey for Pilot testing**

*Adapted from Shaw et al., 2013*

**“Acceptability, appropriateness and effectiveness of mobile SMS intervention to enhance knowledge and behaviors of community people towards dengue prevention in Nepal”**

**Directions:** This tool should be filled in for the participants who received the mobile SMS messages during pre-testing of the intervention messages designed in this study. The assessment should be made for the mobile SMS recipients after sending the designed messages.

**Objectives**: The objective of this assessment is to assess the perception of participants (message recipients) regarding the readability of mobile SMS messages designed for dengue prevention.

**Describe the Reason for the interview:**

*We are here to learn about your experiences with receiving the mobile SMS for dengue prevention activities.*

*Would you be willing to answer some questions regarding your experiences?*

*Please understand that there is no compulsion for you and you are free to decide if you choose not to participate.*

**If no***: Thank them for their time*

**If yes***: Review the purpose of the study and the types of questions that are going to be asked. Remind participants that the interview will be audio-taped.*

**Researcher: Ashmin Hari Bhattarai**

| Code Number | ………………………………… |
| --- | --- |
| Name of Interviewers: | 1. ……………………………….  2………………………………... |

**Attitudes and Behaviors Scale towards Dengue Prevention SMS**

1 = completely disagree, 3 = neutral (neither disagree nor agree), and 5 = completely agree

| **SN** | **Items** | **Score** |
| --- | --- | --- |
| 1 | I feel that receiving the text messages is enjoyable | 1 2 3 4 5 |
| 2 | I feel that receiving the text messages is pleasant | 1 2 3 4 5 |
| 3 | I feel that the text messages are a good source for timely information | 1 2 3 4 5 |
| 4 | I feel that the content of the messages are easy to understand | 1 2 3 4 5 |
| 5 | I feel that the text messages are irritating | 1 2 3 4 5 |
| 6 | I feel that the text messages are too much | 1 2 3 4 5 |
| 7 | Content in the text messages are often annoying | 1 2 3 4 5 |
| 8 | Overall, I like the text messages | 1 2 3 4 5 |

**Closing**

*Thank you for participating in our research study. Please understand that your answers will remain confidential. Give them follow-up contact information such as the telephone number of the PI.*

**Contact person**

If you have any questions regarding this study, you may contact:

Principal Investigator: Ashmin Hari Bhattarai

E-mail: [ashmin.bhattarai@gmail.com](mailto:ashmin.bhattarai@gmail.com)

Phone Number: +977-9855041558 (Nepal) /+6282195049126 (Indonesia)
